# Isolation and Characterization of Homologically Expressed Methanol Dehydrogenase from *Methylorubrum extorquens* AM1 for the Development of Bioelectrocatalytical Systems

**DOI:** 10.3390/ijms231810337

**Published:** 2022-09-07

**Authors:** Tatiana Karaseva, Dmitry Fedorov, Sophia Baklagina, Olga Ponamoreva, Sergey Alferov, Galina Ekimova, Azat Abdullatypov, Liubov Trubitsina, Ildar Mustakhimov

**Affiliations:** 1 Biotechnology Department, Tula State University, Pr. Lenina 92, 300012 Tula, Russia; 2 Laboratory of Methylotrophy, Skryabin Institute of Biochemistry and Physiology of Microorganisms of Russian Academy of Sciences—A Separate Subdivision of Federal State Budget Institution of Science, Federal Research Center (IBPM RAS), Pushchino Scientific Center of Biological Research of Russian Academy of Sciences, Prospekt Nauki 5, 142290 Pushchino, Russia; 3 Laboratory of Ecological and Medical Biotechnology, Tula State University, Friedrich Engels Street 157, 300012 Tula, Russia; 4 Laboratory of Biotechnology and Physiology of Phototrophic Organisms, Institute of Basic Biological Problems Russian Academy of Sciences—A Separate Subdivision of of Federal State Budget Institution of Science Federal Research Center (IBBP RAS), Pushchino Scientific Center of Biological Research of Russian Academy of Sciences, Institutskaya Street 2, 142290 Pushchino, Russia; 5 Laboratory of Microbe Enzymology, Skryabin Institute of Biochemistry and Physiology of Microorganisms of Russian Academy of Sciences—A Separate Subdivision of Federal State Budget Institution of Science, Federal Research Center (IBPM RAS), Pushchino Scientific Center of Biological Research of Russian Academy of Sciences, Prospekt Nauki 5, 142290 Pushchino, Russia

**Keywords:** methanol dehydrogenase MDH, PQQ, His-tag, homologous expression, *Methylorubrum extorquens*, biosensor

## Abstract

(Ca^2+^)-dependent pyrroloquinolinequinone (PQQ)-dependent methanol dehydrogenase (MDH) (EC: 1.1.2.7) is one of the key enzymes of primary C1-compound metabolism in methylotrophy. PQQ-MDH is a promising catalyst for electrochemical biosensors and biofuel cells. However, the large-scale use of PQQ-MDH in bioelectrocatalysis is not possible due to the low yield of the native enzyme. Homologously overexpressed MDH was obtained from methylotrophic bacterium *Methylorubrum extorquens* AM1 by cloning the gene of only one subunit, *mxaF*. The His-tagged enzyme was easily purified by immobilized metal ion affinity chromatography (36% yield). A multimeric form (α6β6) of recombinant PQQ-MDH possessing enzymatic activity (0.54 U/mg) and high stability was demonstrated for the first time. pH-optimum of the purified protein was about 9–10; the enzyme was activated by ammonium ions. It had the highest affinity toward methanol (K_M_ = 0.36 mM). The recombinant MDH was used for the fabrication of an amperometric biosensor. Its linear range for methanol concentrations was 0.002–0.1 mM, the detection limit was 0.7 µM. The properties of the invented biosensor are competitive to the analogs, meaning that this enzyme is a promising catalyst for industrial methanol biosensors. The developed simplified technology for PQQ-MDH production opens up new opportunities for the development of bioelectrocatalytic systems.

## 1. Introduction

The biological conversion of one-carbon compounds is an attractive area of biotechnology because it could lead to significant breakthroughs in the production and utilization of renewable fuels, such as biogas and methanol [1,2]. Methylobacteria are a special group of microorganisms with significant biotechnological potential due to their ability to utilize methanol, methane, and other C_1_-compounds as a source of carbon and energy [3,4,5,6,7]. Methanol dehydrogenases (MDHs) are crucial enzymes for the utilization of methanol as a carbon and energy source in methylotrophy [8]. The engineering of MDHs has thus been actively investigated to enhance methanol conversion [9,10]. One of these interesting biocatalysts is periplasmic pyrroloquinolinequinone (PQQ)-dependent methanol dehydrogenase (EC: 1.1.2.7) [11,12] Its active site includes Ca^2+^ ion. Recently, MDH quinoproteins have attracted a growing interest due to recently found lantanide-dependent isoenzymes of PQQ-MDH (XoxF) [13,14,15,16,17]. However, lanthanide-dependent isoenzymes are less specific to methanol. In all the MDH isoforms, the PQQ cofactor tightly bound to the apoenzyme catalyzes pH-dependent two-electron/two-proton reaction of oxidation of methanol to formaldehyde at relatively low positive potentials, which distinguishes it from other redox cofactors, such as flavins and nicotinamides [18,19].

The native electron acceptor of PQQ-dependent MDH is cytochrome c [20]. MDH is known to manifest in vitro activity in presence of phenazine methosulfate (PMS) or phenazine ethosulfate towards artificial electron acceptors (2,6—dichlorophenolindophenol, 1,6-dichlorophenolindophenol, Wurster’s blue (N,N,N’,N’-tetramethyl-п-phenylenediamine and 2,2′-azino-(-3-ethylbenzthiazoline-6-sulfuric acid)), which are able to receive electrons from the reduced PQQ-enzyme [21]. This fact is the rationale for the application of PQQ-MDH in the development of electrochemical biosensors and biofuel cells (BFCs) [22,23,24,25,26]. In these works, only native methanol dehydrogenases were used.

The application of PQQ-MDH in bioelectrocatalysis is limited by issues related to the purification of homogeneous enzymes. Conventional chromatography methods are used for the isolation and purification of native PQQ-MDH, but they are time-consuming, and they provide low yield because of protein losses at each stage of the multistage process. It is possible to simplify and accelerate the procedure of isolation of homogeneous enzymes by methods of molecular biotechnology based on cloning of the corresponding genes, their expression in superproducer strains, and subsequent purification of the recombinant enzyme by metal chelate affinity chromatography [27]. However, expression of PQQ-MDH in heterologous systems according to conventional cloning protocol leads to isolation of just apoenzyme, because the traditionally used producer strains are genetically incapable of PQQ synthesis. The addition of an expensive PQQ cofactor to the reaction medium for restoration of recombinant MDH activity, as was proposed for glucose dehydrogenase [28], is not promising for further application of the enzyme in bioelectrochemical systems. Huang et al. mentioned that they attempted to express Ln^3+^-dependent alcohol dehydrogenase, classified as XoxF4, XoxF5, in *Escherichia coli* using commercial pET vectors, but the use of such constructs did not result in the production of active enzymes even when cells were grown in the presence of PQQ and Ln^3+^, the natural cofactors, or when the expressed proteins were incubated with these cofactors in attempts to reconstitute active holoenzymes [29]. Their earlier work [30] demonstrated a certain success achieved by a more complicated strategy involving a modified pCM80-PxoxF vector that contained a native promoter for the mxaF gene from *Methylorubrum extorquens* AM1 [31]. The modified vector included a 411 bp-long fragment of synthetic DNA presumably containing the native promoter of xoxF gene of the same organism, synthetic Shine-Dalgarno sequence, unique NcoI restriction site for cloning of the genes of interest, TEV protease cleavage site, and 8-His-tag encoding sequence [30]. However, known methylobacterial strains are also proposed to be used as hosts for obtaining PQQ-MDH [5,30]).

*Methylorubrum extorquens* AM1 (formerly *Methylobacterium extorquens* AM1) is a model strain in methylotrophy studies with a completely sequenced genome [32], and physiological, biochemical, transcriptomic, and proteogenomic aspects of the metabolism of this strain are well-studied. Large and small subunits of MDH are encoded by *mxaF* and *mxaI* gene sequences. PQQ cofactor biosynthesis genes (*pqq*ABC/DE, *pqq*FG) are located in two clusters in another genome locus [33,34,35]. The *mxaF* gene fragment was earlier used as a probe for sequencing of *mxa* genes from *Hyphomicrobium methylovorum* GM2 [36]. Moreover, overexpression of *mxaF* gene was suggested to be used for the elevation of L-serine production by methylotrophic bacteria *Methylobacterium sp.* MB200 [37], but not for obtaining the recombinant protein. Based on the aforementioned data, we proposed to use the homologous protein expression method to obtain active recombinant MDH with a polyhistidine tag for one-step purification of the target enzyme.

The goal of the current work was to obtain active recombinant MDH of *Methylorubrum extorquens* via amplification and cloning of a large subunit gene, *mxaF*, followed by the production of the complete functional protein in a homologous expression system for its further application as a biocatalyst for amperometric mediator biosensor.

## 2. Results

### 2.1. Construction of the Expression Vector

**Figure 1:**
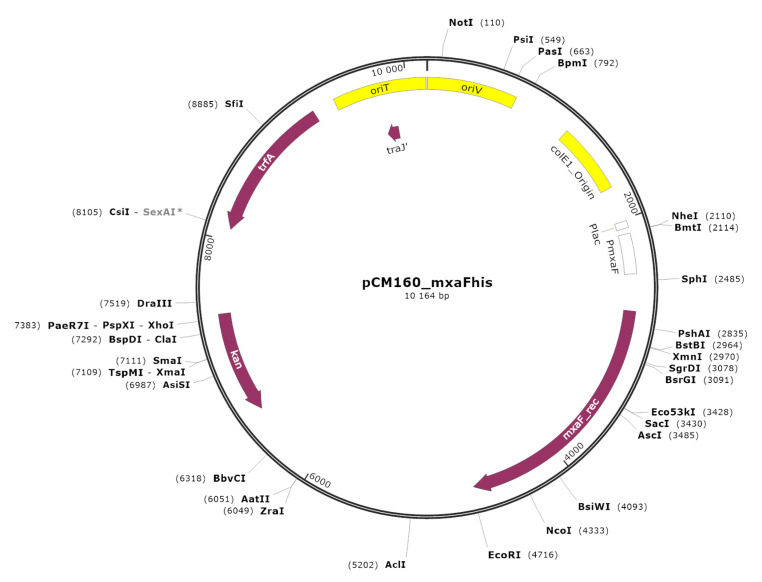
Genetic map of the expression vector with *mxaF* gene from *Methylorubrum extorquens* AM1.

The obtained genetic construct named pCM160::mxaF is presented in Figure 1.

Amplification of DNA fragment from *Methylorubrum extorquens* AM1 containing mxaF gene (GenBank locus tag: MexAM1_META1p4538, chromosome accession: NC_012808) was carried out using specific primers and the genomic DNA of the strain as a template. The resulting PCR fragment containing full-length *mxaF* gene ORF with encoded leader peptide and C-end fusion with His6-tag was cloned into mobilizable vector pCM160 [33] under the control of MDH promoter (PmxaF) from AM1 strain. Construct pCM160::mxaF was used for the transformation of competent *E. coli* TOP10 cells. PCR-screening of the clones for the presence of recombinant plasmid showed an insertion of the corresponding length in 60% of the samples (Figure S1). After isolation of plasmid DNA, PCR and restriction analysis was carried out for plasmid examination (Figure S2). The analyses confirmed the presence of the insertion of the desired size and correct arrangement of the construct. The obtained recombinant plasmid was checked for the absence of mutation by direct sequencing. To obtain the active recombinant MDH, the enzyme was expressed in the host organism, *M. extorquens* AM1.

### 2.2. Expression and Purification of the Recombinant MDH

**Figure 2:**
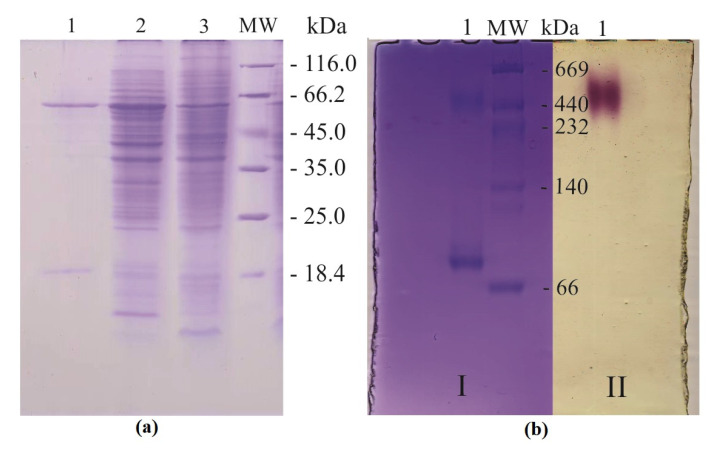
(**a**) SDS-PAAG electrophoresis of the purified recombinant protein MDH-His_6_-tag and supernatant of recombinant and wild-type strains of *M. extorquens*. The concentration of the separating gel was 12%, SDS was added to 0.1% concentration. 1—purified enzyme, 2—supernatant of *M. extorquens* pCM160::mxaF, 3—supernatant of *M. extorquens* AM1, MW—molecular weight markers (Unstained Protein MW Marker, Thermo Fisher Scientific, USA). (**b**) Native PAAG with 5–12% concentration gradient. (I) 1—purified enzyme, MW—molecular weight markers (High MW Native Marker Kit, GE Healthcare, USA); (II) Visualization of MDH activity. Methanol dehydrogenase activity was detected using nitroblue tetrazolium and phenazine methosulfate in the presence of 20 mM methanol and 15 mM ammonium chloride.

His_6_-tagged recombinant protein (MDH-His_6_-tag) was isolated by affinity metal chelate chromatography to an electrophoretically homogeneous state. Denaturing SDS-PAAG electrophoresis of the purified protein revealed the presence of a large subunit (α) (MW around 66 kDa) and a small subunit (β) (around 18 kDa) in the collected fractions (Figure 2a).

The data are in accordance with the value calculated from translated nucleotide sequence for *mxaF* gene for the large subunit of MDH (68434 Da) (Refseq/GenBank NC_012808/MEXAM1_RS21435). When comparing the electropherograms of the supernatants, elevated production of α-protomer of MDH in *M. extorquens* pCM160::mxaF is clearly seen (Figure 2a, band 2), which confirms the functioning of the inserted *mxaF* gene.

Native electrophoresis of the purified protein preparation showed the presence of two enzyme forms, heterodimeric (αβ) and multimeric (presumably α_6_β_6_) with MW around 75 and 450 kDa, respectively (Figure 2b, I). It should be noted that the molecular weight of the low molecular weight fraction of the recombinant protein determined by gel filtration was 75 kDa, which is in accordance with native electrophoresis data. A surprising result was the presence of enzymatic activity only in oligomeric form (Figure 2b, II). The results of MDH-His_6_-tag purification are listed in Table 1.

The highest specific activity was observed in the first fraction of the elution buffer. The enzyme was purified 7-fold up to the specific activity of 3.74 U/mg protein. The activity of the recombinant MDH-His_6_-tag in the supernatant equaled 0.54 U/mg, which was higher compared to the native MDH from wild-type strain *M. extorquens* AM1 comprising 0.34 U/mg. The yield of the purified protein was 36%.

### 2.3. Characterization of the Recombinant MDH

An absorption maximum at 350 nm and a wide absorption shoulder to 410 nm were observed in the absorption spectrum of the purified preparation of MDH-His_6_-tag.

**Figure 3:**
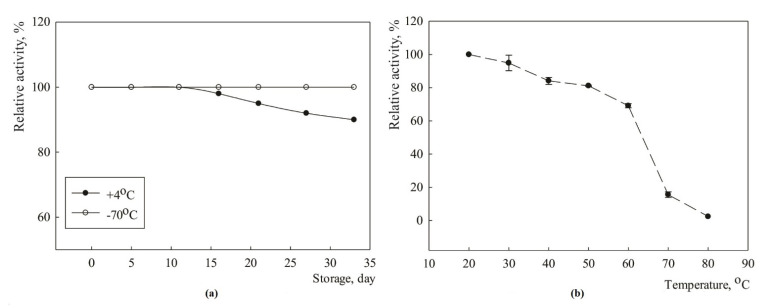
Stability of MDH-His_6_-tag (1.3 mg/mL): (**a**) Upon storage in 25 mM potassium phosphate buffer solution (pH 6.5) at +4 °C without stabilizing agents, and at −70 °C with 50% glycerol supply; (**b**) Thermostability of MDH-His_6_-tag at pH 8.0. Activity of the non-heated enzyme was defined as 100%.

The activity of the purified MDH-His_6_-tag preparation was decreased by only 10% after 30-day storage at +4 °C, whereas the enzyme remained fully active upon storage at −70 °C (Figure 3a).

The thermostability study revealed that in 20 min of incubation in a temperature range from 20 to 50 °C the enzyme lost up to 20% of its activity, whereas complete denaturation was observed at 80 °C (Figure 3b).

The enzyme activity decrease rate is around 0.5% × min^−1^ at 40 °C and 50 °C, and around 1% × min^−1^ at 60 °C (Table 2).

The recombinant MDH was stable in a pH range from 7 to 11 (Table 3), which is in agreement with data for native MDH from *M. extorquens* AM1 [38].

With the increase of the primary alcohol carbon chain length, the apparent value of K_M_ of the recombinant enzyme was also increased, whereas maximal reaction velocity did not change significantly (Table 4).

### 2.4. Biosensor Based on the Recombinant MDH

After immobilization of MDH-His_6_-tag onto the surface of the electrode containing 30% hydroxyl apatite and 10% ferrocene in the graphite paste, the response of the biosensor to methanol was elevated by more than 2 times compared to paste without hydroxyl apatite under identical measurement conditions. The mean value for the obtained responses of the biosensors to 0.05 mM methanol (the medium value for the linear range) was 1600 ± 100 nA for the hydroxyl apatite-modified electrode, and the relative standard deviation for 15 consequent measurements was 7.1% (*p* = 0.95).

Specificity of the recombinant MDH-His_6_-tag-based biosensor in presence of ferrocene revealed the dependence of the decrease of sensor responses (nA) on the increase of carbon chain length: methanol, 19,600 ± 100; ethanol, 13,000 ± 2000; butanol-1, 9200 ± 700; 3-methylbutanol-1, 7000 ± 100; 2-methylpropanol-1, 2400 ± 200 (*p* = 0.95; *n* = 3). The highest responses of the biosensors were achieved for such substrates as methanol, formaldehyde (17,000 ± 2000 nA), and ethanol, which is in agreement with the substrate specificity of the enzyme (Table 4).

To determine the parameters of the biosensor (sensitivity coefficient and detection limit), the linear segment of the calibration dependence was used. The linear range for the detected concentrations was from 0.002 to 0.1 mM for methanol, detection limit was equal to 0.7 µM CH_3_OH, and the duration of a single measurement comprised 5 min.

## 3. Discussion

One of the recent reviews on the expression of halobacteria proteins notes the potential of methods for the homologous expression of proteins containing a specific cofactor, such as metalloproteins [39]. This method can also be used to obtain PQQ-methanol dehydrogenase. At present time, expression vectors based on a strong promoter of the methanol dehydrogenase gene from *M. extorquens* (P_mxaF_) are used for cloning different proteins into methylotrophic bacteria [31]. In the present work, one of the known vectors was used, a shuttle vector pCM160 containing a constitutive promoter of MDH from *M. extorquens* (318 bp) which is capable of replication in both methylotrophic bacteria and *E. coli* [31]. This is important for the development of an efficient technique for homologous cloning of MDH. (Table 5). The limiting stages in the technique of homologous expression of active recombinant MDH are the stages of *Methylobacterium* cell cultivation on liquid and solidified media, which is due to their growth physiology. It should be noted that the recombinant strain *Methylorubrum extorquens* pCM160::mxaF does not differ from the initial AM1 strain by its growth properties. The other stages, including *mxaF* gene cloning, sequence analysis of the plasmid construction, transformation of competent *E. coli* Top10 cells, clone selection, conjugative transfer into *M. extorquens* and purification of recombinant MDH do not take more than a week. The overall process from PCR amplification to isolation of the desired enzyme can be carried out for 23 days. The time of isolation of active PQQ-MDH could be reduced when using the ready recombinant strain containing the modified plasmid.

According to proteomic studies ([7], MDH is one of the most abundant proteins in AM1 strains during methylotrophic and other variants of heterotrophic growth. Since the mxaF gene is located in one cluster with mxaI, the small subunit should be synthesized in a comparable amount [11]. Despite the fact that PQQ biosynthesis genes are located in other separate clusters, the cofactor appears to be produced in sufficient amounts even upon the insertion of extra copies of the mxaF gene on the plasmid. All of the above allows to isolate active recombinant holoenzyme.

It was possible to increase the yield of recombinant MDH compared to native MDH, despite the fact that only one subunit of the *mxaF* gene was cloned. In the majority of papers on PQQ-MDH, the reported yield did not exceed 29% after several consequent stages of chromatography [40,41,42,43,44]. This is additional proof of elevation of the expression of functionally active MDH. Thus, the one-step technique elaborated in our work could increase the yield and reduce the time required for enzyme isolation.

The absorption spectrum of MDH-His_6_-tag is identical to the spectra of PQQ-dependent native MDHs of methylobacteria [25,45,46]. It should be noted the majority of native MDHs characterized before are heterodimers [47] or heterotetramers [41,48]. The active site with non-covalently bound prosthetic groups, PQQ and Ca^2+^ ion, is located in the large α-subunits *M. extorquens* AM1 with MW ~66 kDa [20,40,48], ~62 кДa [49]. The small subunit (7.5–10 kDa) is not present in other quinoproteins, and its role remains unclear [40,48]. Heterohexameric form (α_6_β_6_) of the recombinant MDH possessing enzymatic activity (a trimer of canonical α_2_β_2_ form) was revealed for the first time.

The addition of an activator, NH_4_^+^, is required for manifestation of the MDH-His_6_-tag activity, as well as for native PQQ-MDHs [40,42,47,48]. In presence of ammonium chloride, MDH-His_6_-tag is capable of oxidizing primary alcohols and formaldehyde with the highest affinity towards methanol, similar to other MDHs [43,44,47,50].

Storage stability is one of the important characteristics of enzymes for manufacturing enzymatic biosensors. Compared to the native MDHs [47,50], whose activity decreased by around 20% upon storage at +4 °C, the recombinant MDH was characterized by higher stability. It is intriguing because one paper mentioned that the thermal stability of the recombinant MDH is comparable to that of the native enzyme [47]. The relatively high stability of the recombinant MDH is an important aspect for using the enzyme in bioelectrocatalytic systems.

**Figure 4:**
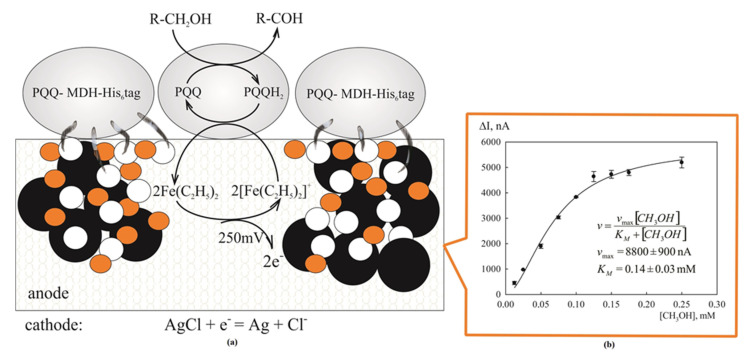
Process occurring upon oxidation of primary alcohols under the action of PQQ-cofactor-containing MDH-His_6_-tag in presence of ferrocene (**a**). Dependence of amperometric MDH-His_6_-tag -based mediator biosensor response (∆I) on methanol concentration (**b**). Biosensor operation conditions: 50 mM potassium phosphate, pH 8.0, supplied with 15 mM NH_4_Cl;.

The application of native MDH as a base for biosensors was described in several papers. Immobilized MDH from *Methylobacterium extorquens* AM1 was applied for amperometric detection of ammonia in presence of phenazine methosulfate [23] and methanol [24]. In another work [25], MDH from *Methylobacterium nodulans* was immobilized on the graphite paste electrode for amperometric determination of methanol concentration in presence of the electron transport mediator, ferrocene. The electrochemical oxidation of methanol and formaldehyde catalyzed by Eu^3+^-MDH with its own native cytochrome electron acceptor, c*_GJ_*, co-adsorbed by chitosan biopolymer, was studied in the work [26]. It is known that the most sensitive biosensing systems based on PQQ-dehydrogenases are developed using ferrocene and its derivatives as electron transfer mediators [51,52]. The preparation of the MDH-His_6_-tag was used for the development of amperometric biosensor based on graphite paste electrode and ferrocene. To immobilize the recombinant MDH, a method for modification of graphite paste by hydroxyl apatite elaborated earlier [25] was used, which improves the adsorption of the enzyme onto the surface of the hydrophobic electrode. The process occurring upon oxidation of primary alcohols under the action of the PQQ cofactor-containing MDH-His_6_-tag is drawn in Figure 4. The active site of the enzyme interacts with ferrocenium ion, reducing it to ferrocene. Under the external potential applied to the working electrode, ferrocene is oxidized to ferrocenium ion and gets involved in the new interaction cycle.

The dependence of current on methanol concentration had a hyperbolic character (R^2^ = 0.96) (Figure 4b), which is in accordance with typical enzymatic kinetics [53]. This dependence was approximated by a Michaelis–Menten-like equation (*p* = 0.95; *n* = 3). The high response of the biosensor to formaldehyde is presumably determined by the non-specific oxidation of the hydrated formaldehyde species (geminal diol) by MDH [54]. Being simple and economical in fabrication, MDH-His_6_-tag-biosensor does not give way to other methanol biosensors [55,56,57], and even surpasses them in some analytical and metrological characteristics.

Thus, the recombinant MDH could serve as an effective biocatalyst for amperometric mediator biosensors for methanol, and the characteristics of the invented biosensor are enough to compete with the analogs.

## 4. Materials and Methods

### 4.1. Objects of the Study and Cultivation Conditions

The presented method of homologous expression of active recombinant MDH with His_6_-tag consists of the stages listed in Table 5.

Aerobic methylotrophic bacteria from the collection of the laboratory of methylotrophy of Skryabin Institute for Biochemistry and Physiology of Microorganisms (Russia). Were used in the study (strain *Methylorubrum extorquens* AM1 CIP 106,787 = DSM 6343 = VKM B-2191), as well as *Escherichia coli* Top 10 and S17-1.

Bacterial strains and plasmids are listed in Table 6.

*M. extorquens* was cultivated in 100 mL Erlenmeyer flasks in «K» medium with the following composition (g/L): KH_2_PO_4_—2, (NH_4_)_2_SO_4_—2, NaCl—0.5, MgSO_4_ ∙7H_2_O—0.025, FeSO4·7H_2_O—0.002, pH 7.2 supplied with 1% methanol (*v*/*v*). The cultures were grown in thermostabilizable shakers (180 rpm, 29 °C). A solid medium for *M. extorquens* colony selection contained 1.5% agar

*E. coli* strains were grown at 37 °C in liquid or solidified (1.5% agar) LB medium with kanamycin antibiotic added up to 50 µg/mL.

### 4.2. Construction of Vector for Expression/Production of the Recombinant Protein

Gene *mxaF* from *M. extorquens* (GenBank locus tag: MexAM1_META1p4538, chromosome accession: NC_012808) (2248 bp) was amplified using the designed primers: forward, 5′-CTAATGCATGCGCCCGTTGACGACAACGGTG-3′, and reverse, 5′-TAGAATTCTCAGTGGTGGTGGTGGTGGTGCTTGGCGGCCGACTTCCACTC-3′. The reverse primer contains a sequence encoding poly-His-tag (shown by italic). The primers’ sequences contained PaeI (forward) and EcoRI (reverse) restriction endonucleases sites (underlined in the sequences). The insertion of this sequence allows obtaining a protein with a C-terminal His-tag.

The genomic DNA of AM1 strain was used as a template for amplification. Obtained 2248-bp PCR product was treated with PaeI and EcoRI endonucleases and cloned into pCM160 expression vector cleaved at the same sites. Thus, a recombinant plasmid pCM160::mxaF was obtained.

### 4.3. Conventional Methods

#### 4.3.1. Polymerase Chain Reaction (PCR)

PCR was performed on MJ Mini thermal cycler (Bio-Rad, Hercules, CA, USA) in 20 µL of a mixture containing 0.26 pmol/µL of each primer, 2 µL of 10× PCR buffer solution, 3% of dimethyl sulfoxide (DMSO) (Sigma-Aldrich, Burlington, MA, USA), 0.075 mmol/L of deoxyribonucleotide triphosphates (Thermo Fischer Scientific, Waltham, MA, USA), 0.5 µL of KAPA Hi-Fi DNA polymerase (Roche, Indianapolis, IN, USA).

To amplify the *mxaF* gene from *M. extorquens*, the following time–temperature profile was used: preliminary denaturation—94 °C, 5 min; 32 cycles of denaturation (94 °C, 30 s), annealing (64 °C, 20 s), elongation (72 °C, 3 min 30 s); final elongation (72 °C, 5 min).

#### 4.3.2. Treatment of DNA with Restriction Endonucleases

Hydrolysis of PCR amplicons and vectors with different endonucleases was conducted according to the manufacturer’s instructions (Thermo Fischer Scientific, Waltham, MA, USA), using recommended buffer solution and corresponding temperature conditions for each enzyme. DNA fragments were separated by electrophoresis in 1% agarose gel. DNA molecular weight markers (GeneRuler DNA Ladder Mix, Thermo Fischer Scientific, Waltham, MA, USA) were used for the estimation of the size of DNA fragments. After electrophoresis, the DNA fragments were purified from gel using QIAquick Gel Extraction Kit (Qiagen, Hilden, Germany) according to the manufacturer’s recommendations.

#### 4.3.3. Ligation of DNA Fragments

The ligation reaction was performed in 10 µL volume containing 1 µL of 10× ligation buffer (SibEnzyme, Novosibirsk, Russia), 50–100 ng of vector DNA and 3–5× molar excess of the cloned DNA fragment, 10 units of T4 DNA ligase (SibEnzyme, Novosibirsk, Russia). Sticky-end ligation was carried out for 10–16 h at 4 °C. The reaction was stopped by enzyme inactivation for 15 min at 65 °C, and the competent *E. coli* TOP10 cells were transformed with the ligation mixture.

#### 4.3.4. Isolation of Plasmid DNA and Transformation of Competent Cells

Isolation of plasmid DNA, cloning, and transformation of competent cells was performed according to conventional techniques [59].

#### 4.3.5. Conjugative Plasmid Transfer

Conjugative plasmid transfer from *E. coli* S17-1 cells to recipient strain AM1 was carried out on Petri dishes with solidified «K» medium (1.5% agar) containing 2% methanol (*v*/*v*) and 0.5% LB medium for 24 h. Donor to recipient ratio was 1:1 by cell number. After the 24-h incubation, the cultures were washed from the filters with 2 mL of «K» medium, and the dilutions were plated onto the selective solid «K» medium with kanamycin (50 µg/mL), nalidixic acid (100 µg/mL), and 2% methanol (*v*/*v*) as a sole source of carbon and energy. Transconjugants were analyzed by PCR and sequencing for the presence of the plasmids and the *mxaF* gene.

### 4.4. Isolation of Recombinant Methanol Dehydrogenase by Metal Chelate Affinity Chromatography

Transconjugants of methylobacteria carrying the recombinant plasmids were grown in 200 µ of K medium supplied with 50 µg/mL kanamycin and 1% methanol at 29 °C. To purify the recombinant proteins, cells (0.8 g) were collected by centrifugation, resuspended in 2.5 mL of cold lysis buffer (20 mM Tris-HCl, 500 mM NaCl, 5 mM imidazole, pH 8.0), and disintegrated by a sonicator (Misonix Ultrasonic Liquid Processor S-4000/CL5 ultrasonic disintegrator, UK) (16 × 30 s with 30 s interval). The cell lysate was centrifuged at 15,000 rpm for 30 min at 4 °C, and the supernatant was applied to a 0.5 mL Ni^2+^-NTA-agarose column (Qiagen, Hilden, Germany). After washing with washing buffer (20 mM Tris-HCl, 500 mM NaCl, 60 mM imidazole) the bound protein was eluted from the column with the analogous buffer containing 200 mM imidazole. The protein spectrum of the collected fractions was analyzed by SDS-PAAG-electrophoresis. All the purification steps were carried out at 4 °C.

### 4.5. Methanol Dehydrogenase Activity Measurement

MDH activity was determined spectrophotometrically at 600 nm (ε = 1,9 × 10^4^ M^−1^cm^−1^) at 30 °C, according to the technique by Kuznetsova and co-workers [47].

The amount of MDH catalyzing conversion of 1 µmol of methanol into formaldehyde in 1 min at pH 9.0 and t = 30 °C was taken as activity unit.

Determination of kinetic parameters of MDH with different substrates (methanol, ethanol, butanol, amyl alcohol, formaldehyde) was carried out in the concentration range 0–10 mM, the plots were built using 13 different substrate concentrations. The measurements were performed in triplicates.

pH-stability of the purified preparation of MDH-His6-tag was analyzed in pH range from 4 to 11. For this purpose, the enzyme was incubated in buffer solutions (Acetate-NaOH, KH_2_PO_4_-NaOH, Tris-HCl, Glycine-NaOH, Na_2_HPO_4_-NaOH) with the corresponding pH values for 40 min at 30 °C. Then the residual activity was measured in standard conditions at pH = 9.0. To test the thermal stability of the enzyme, MDH samples were incubated at temperatures from 20 to 80 °C for 20–60 min, after that the residual activity was determined in a standard reaction mixture at 30 °C.

### 4.6. Electrophoresis in Polyacrylamide Gel

The purity of protein preparations and subunit composition was determined by denaturing electrophoresis in PAAG with 0.1% sodium dodecyl sulfate (SDS) according to the Laemmli method. The concentration of the separating gel was 12%. Electrophoresis was carried out at 60 V for concentrating gel and 100 V for separating gel in 0.75 mm PAAG slabs at room temperature. Separation was carried out in Tris-glycine buffer system, pH 8.3. The samples were prepared in 0.0625 M Tris-HCl buffer, pH 6.8, supplied with 2% SDS, 0.001% bromophenol blue, 10% glycerol, and 5% β-mercaptoethanol. The samples were boiled for 10 min for protein denaturation. Standard protein ladder PageRuler Prestained Protein Ladder (Thermo Fischer Scientific, Waltham, MA, USA) with molecular masses from 14 to 116 kDa was used for protein mass determination.

The quaternary structure of the native enzyme was determined according to the results of native electrophoresis in polyacrylamide gel (PAAG) with a concentration gradient from 5 to 12%. The protein bands were stained according to Fairbanks et al. (1971) with some modifications using Coumassie R-250 and also stained by activity in presence of nitroblue tetrazolium and phenazine methosulfate according to the known technique [60].

### 4.7. Protein Molecular Mass Determination

The molecular mass of the native protein was determined by gel filtration on Sephacryl S-200 column (1.5 × 60 cm, Pharmacia, Uppsala, Sweden) calibrated using standard protein set MWGF200 S8445 (Sigma-Aldrich, Burlington, MA, USA) containing 150 mM KCl. As the elution buffer, 20 mM Tris-HCl, pH 7.5, was used. The flow rate was adjusted to 0.2 mL/min. The protein content was assessed by the Bradford method using standard BSA solutions.

### 4.8. Electrochemical Measurements

Fabrication of working electrodes and electrochemical measurements were conducted according to the described technique [25]. The electrode was filled with graphite paste containing 10% ferrocene and 30% hydroxyl apatite (*w*/*w*). The enzyme (0.26 units) was applied onto the electrode surface and dried for 30 min. The composition of the measurement buffer solution was as follows: 50 mM potassium phosphate, pH 8.0, supplied with 15 mM NH_4_Cl. The electrode was stored at 4 °C in 50 mM potassium phosphate buffer solution, pH 7.0. The measurements were taken in triplicates.

## 5. Conclusions

The method for homologous expression of the recombinant PQQ-MDH with polyhistidine tag elaborated in the present study allows obtaining an active enzyme in quite a short time. Overexpression of just one subunit, *mxaF*, was sufficient to achieve overproduction of the MDH holoenzyme. One-stage purification of the recombinant enzyme is more economically feasible, which is especially important when designing the consumables for biosensor analytics. The recombinant enzyme does almost not differ from the native MDH of *Methylorubrum extorquens* AM1 in its catalytic properties, but it has higher stability. The developed simplified technology for producing recombinant PQQ-MDH opens up new opportunities for the development of methanol biosensors and biofuel cells. A similar strategy, based on the overexpression of just one subunit and its modification by His-tag insertion, might be used in future works on the enzymes of this class from other methylobacteria.

## Figures and Tables

**Table 1:** Purification of MDH-His_6_-tag from *M. extorquens* pCM160::mxaF_M.ext.

Purification Step	Specific Activity, Units/mg	Activity, Units/mL	Protein, mg/mL	Total Activity, Units	Total Protein, mg	Yield, %	Purification Degree
*Supernatant*	0.54	1.04	1.93	2.60	4.82	100.0	1.0
*MDH-* His_6_-tag	3.74	0.93	0.25	0.93	0.25	35.6	7.00

**Table 2:** Thermal stability of MDH-His_6_-tag measured by residual activity, %.

Temperature, °C	Incubation Time, min			
	20	30	45	60
40	82.6	71.3	69.4	61.1
50	81.4	64.7	64.7	64.1
60	68.3	43.7	49.1	29.9

**Table 3:** The used buffer solutions and corresponding MDH-His_6_-tag activities.

Buffer Solution, 0.1M	pH	Activity, %
Acetate-NaOH	4	56.1 ± 0.6
Acetate-NaOH	5	77 ± 3
KH_2_PO_4_-NaOH	6	84 ± 2
KH_2_PO_4_-NaOH	7	92 ± 3
Tris-HCl	8	92 ± 2
Tris-HCl	9	100
Glycine-NaOH	10	93 ± 0.2
Na_2_HPO_4_-NaOH	11	91 ± 0.1

**Table 4:** Michaelis–Menten parameters of MDH-His_6_-tag in reactions with different substrates (*p* = 0.95; *n* = 3). Reaction rates were measured at 30 °C, pH 9.0.

Substrate	Apparent *K_M_*, mM	Vmax, U/mg Protein	Activity, %
Methanol	0.36 ± 0.07	4.2 ± 0.2	100
Ethanol	0.61 ± 0.06	2.9 ± 0.1	79
Butanol-1	1.9 ± 0.2	2.7 ± 0.1	71
Amyl alcohol	1.4 ± 0.1	2.8 ± 0.1	67
Formaldehyde	n.a.	n.a.	91

**Table 5:** The sequence of stages of obtaining the recombinant MDH by homologous expression method.

Stage of Recombinant MDH Purification	Time Required, Days
Designing and purchasing primers	1
2. Amplification of *mxaF* from *M. extorquens* by PCR method	1
3. Purification of PCR product (amplicon)	
4. Restriction of pCM160 plasmid and amplicon by PaeI and EcoRI enzymes	1
5. Ligation of the fragments and obtaining the recombinant plasmid pCM160/mxaF	
6. Transformation of *E.coli* Top10 by pCM160mxaF plasmid constructions	1
7. Plasmid isolation and sequencing	1 (plus shipping time in case of sequencing in other institutions)
8. Clone selection	2
9. Conjugative transfer into *M. extorquens*	3
10. Selection on kanamycin medium	7
11. Cultivation of biomass in bulk volume and protein expression	7
12. Enzyme purification and activity determination	1
Total time, days:	25

**Table 6:** Bacterial strains and plasmids.

Strain or Plasmid	Specification	Reference
*Escherichia coli*		
S17-1	*thi pro recA hsdR* [RP4-2Tc::Mu-Km::Tn7] Tpr Smr	[58]
TOP10	F^–^ *crA*Δ*(mrr + hsdRMS + mcrBC)* ϕ*80lacZ*Δ*M15*Δ*lacΧ74recA1araD139* Δ*(ara + leu)7697 galU galK rpsL (Strr) endA1 nupG* λ-	Invitrogen, Carlsbad, CA, USA
*Methylo* *rub* *rum extorquens*		
AM1	Wild-type strain	
pCM160::mxaF	Recombinant strains containing pCM160mxaF, K_m_^r^ plasmid	This work
Plasmids		
pCM160	Mobilizable vector for protein expression in *M. extorquens* under control of *mxaF* gene promoter, K_m_^r^	[31]
pCM160::mxaF	pCM160 containing PaeI/EcoRI-fragment with *MxaF* gene from *M. extorquens* AM1, K_m_^r^	This work

## Supplementary Materials

The following supporting information can be downloaded at: https://www.mdpi.com/article/10.3390/ijms231810337/s1.
