# Supplementary material for: Isolation and Characterization of Homologically Expressed Methanol Dehydrogenase from Methylorubrum extorquens AM1 for the Development of Bioelectrocatalytical Systems
Source: Int J Mol Sci. 2022 Sep 7;23(18):10337. doi: 10.3390/ijms231810337 (PMC9499683; doi:10.3390/ijms231810337)
Supplement: Supplementary file 1 [file ijms-23-10337-s001.zip › Supplementary materials_Karaseva.pdf]

Supplementary materials

2245 bp

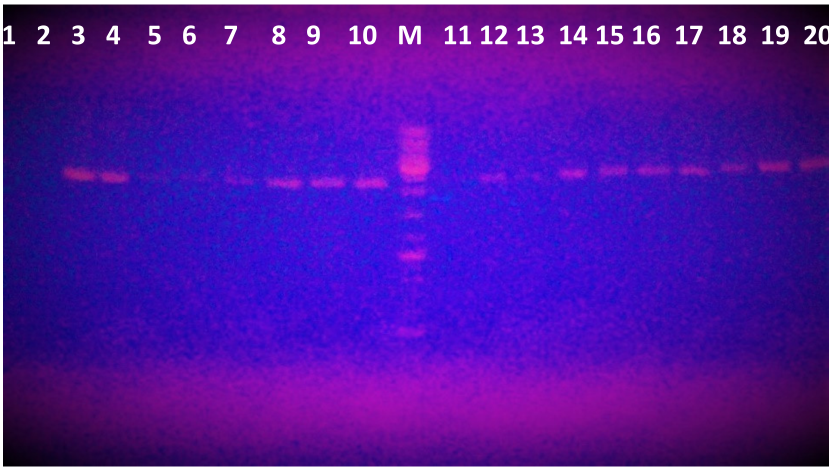

Figure S1. Electrophoregram of PCR products from the plasmid DNA of Top10::pCM160mxaF clones.

2245 bp

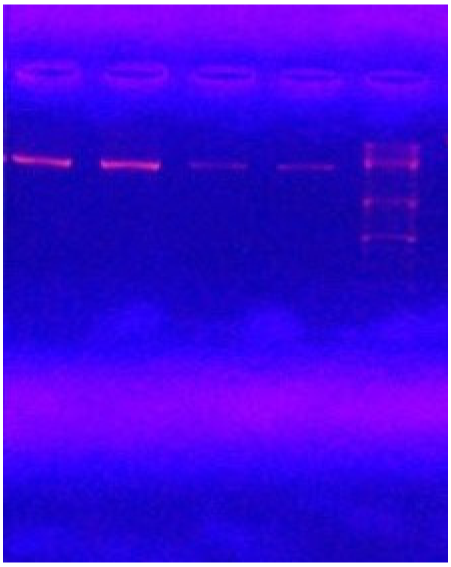

Figure S2. Electrophoregram of PCR products from the plasmid DNA of Top10::pCM160mxaF clones.
